# A Rare Case of Transvaginal Sigmoid Evisceration in a Patient with Recurrent Pelvic Organ Prolapse

**DOI:** 10.3390/jcm14207224

**Published:** 2025-10-13

**Authors:** Belita Opene, Erin Mowers, Bestoun Ahmed, Mary F. Ackenbom, Gnankang Sarah Napoé

**Affiliations:** 1 Department of Obstetrics, Gynecology and Reproductive Sciences, University of Pittsburgh Medical Center, Pittsburgh, PA 15213, USA; 2 Department of Obstetrics and Gynecology, University of Illinois Peoria College of Medicine, Peoria, IL 61605, USA; emowersmdphd@gmail.com; 3 Department of Surgery, University of Pittsburgh Medical Center, Pittsburgh, PA 15219, USA; 4 Department of Obstetrics and Gynecology, University of Michigan, Ann Arbor, MI 48109, USA; 5 Magee-Womens Research Institute, Pittsburgh, PA 15213, USA

**Keywords:** large bowel evisceration, vaginal cuff dehiscence, pelvic organ prolapse, vaginal vault prolapse

## Abstract

Large bowel evisceration is a rare but morbid presentation that requires timely diagnosis and management. We present the case of a 67-year-old woman with a history of recurrent pelvic organ prolapse (notably with a history of prior hysterectomy, mesh-augmented sacrocolpopexy, and transvaginal Uphold™ mesh placement). She presented with the subjective report of subacute worsening of her prolapse leading to urinary retention managed with a Foley catheter and an irreducible vaginal mass prompting evaluation. Clinical evaluation revealed bowel contents in the vagina with subsequent initiation of intravenous antibiotics, diagnostic laparoscopy converted to exploratory laparotomy, and resection of sigmoid and upper rectum with creation of left ileal end colostomy. Common risk factors for bowel evisceration include older age, postmenopausal status, history of pelvic surgery, and pessary use. In a patient with subacute worsening of prolapse and pain with the above risk factors, bowel evisceration should be considered and ruled out.

## 1. Introduction

Transvaginal evisceration of bowel refers to the protrusion of intra-abdominal contents through a vaginal vault defect or full-thickness separation of the anterior and posterior vaginal cuff. Transvaginal evisceration is a very rare event with a reported incidence of 0.032–1.2% [1]. Most transvaginal evisceration commonly involves the small bowel [1]. Large bowel evisceration is rare, with only a few case reports [2,3,4,5]. This event is a surgical emergency. Early recognition and timely evaluation are warranted to prevent morbidity and to rule out other acute differential diagnoses.

Here, we present a case of transvaginal evisceration of the sigmoid colon in a patient with known recurrent vaginal vault prolapse who initially believed she was experiencing worsening prolapse.

## 2. Case Presentation

The patient is a 67-year-old female with a history of pelvic organ prolapse, urgency urinary incontinence, and an extensive history of pelvic surgeries who presented for a visit at the urogynecology outpatient office with the report of a 3-week history of initially reducible prolapsing vaginal mass that became non-reducible. She described worsening prolapse leading to urinary retention and subsequent Foley catheter placement 2 weeks prior to her presentation while receiving care in a nursing facility for management of right lower extremity cellulitis with intravenous antibiotics. The patient reported that she had new-onset diarrhea and fecal incontinence at the time of her evaluation, with normal flatus. The patient’s surgical history was significant for a total abdominal hysterectomy and sacrocolpopexy with anterior and posterior repair using hernia mesh in 2012. In 2015, the patient experienced both mesh exposure necessitating transvaginal mesh removal and subsequent vaginal vault prolapse recurrence managed with mesh-augmented bilateral sacrospinous ligament suspension with concomitant anterior colporrhaphy. Following a second recurrence of her prolapse after the vaginal mesh procedure, the patient was initially managed with a pessary for ongoing stage III vaginal vault prolapse and persistent mixed urinary incontinence. However, pessary management was eventually discontinued due to repeated visits for vaginal erosions and bleeding.

## 3. Physical Examination

**Figure 1:**
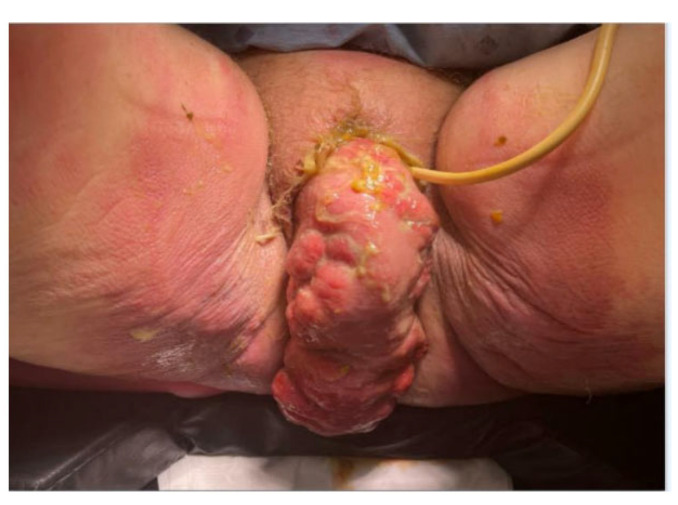
Transvaginal evisceration of the sigmoid colon at presentation with a Foley catheter in situ.

Her exam in the office prompted transfer to the emergency department (ED). In the ED, the patient was well-appearing and hemodynamically stable. She was afebrile, normotensive, and non-tachycardic. Her exam revealed a large prolapsing vaginal mass measuring approximately 16–18 cm in diameter. The mass was geographic and edematous in appearance, firm, tender to palpation, and not easily reducible, with a tan-green discharge noted along with nodular, excoriated vaginal mucosa. Bowel contents were palpable within the prolapse, distinct from the vaginal walls, with referred abdominal tenderness (Figure 1). Due to the size of the prolapse and patient discomfort, assessment of the vaginal epithelium integrity and thickness was limited. The rectum was also difficult to visualize. No exposed mesh was seen. There was a Foley catheter in place, draining clear urine.

## 4. Laboratory and Imaging

**Figure 2:**
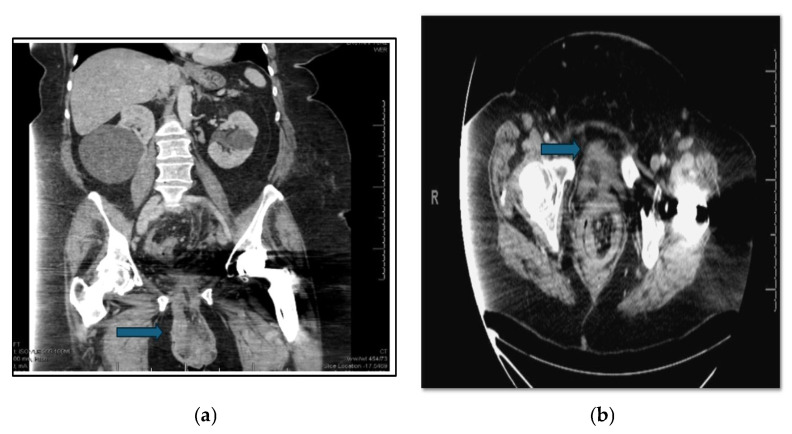
(**a**) Axial CT scan of the abdomen and pelvis demonstrates prolapsed loops of large bowel in the vagina. The blue arrow indicates eviscerated bowel. (**b**) Coronal view CT abdomen and pelvis scan, and blue arrow showing the prolapsing loops of large bowel.

Laboratory results revealed leukocytosis of 13,800 cells/µL, normal lactic acid with subsequent elevation eight hours later to 2.3 mmol/L, hypokalemia with a potassium of 2.6 mmol/L, and acute kidney injury with creatinine 1.1 mg/dL. Urinalysis was consistent with a urinary tract infection, and a urine culture was ultimately positive for greater than 100,000 cfu/mL of Escherichia coli and 50,000–100,000 cfu/mL of Klebsiella pneumoniae. A CT scan of the abdomen and pelvis revealed a large vaginal prolapse containing a segment of the distal sigmoid colon twisted upon itself, consistent with a volvulus. Associated findings included pericolonic fat stranding, bowel wall thickening, and hyperenhancement, suggestive of ischemia. Additionally, the inferior bladder appeared herniated within the prolapse (Figure 2a,b).

## 5. Surgical Management

Fluid resuscitation and intravenous administration of broad-spectrum antibiotics- ceftriaxone and metronidazole were initiated, given high suspicion of evisceration, and the patient was admitted to the hospital. The patient was counseled on surgical options, including the risks and benefits, given her extensive pelvic surgical history. However, the patient desired to remain sexually active and declined pursuing colpocleisis, which was not performed. The patient was taken emergently to the operating room for surgical intervention. Initial diagnostic laparoscopy with adhesiolysis was converted to an exploratory laparotomy due to extensive bowel edema and intraoperative adhesions, with subsequent confirmation of vaginal vault dehiscence and evisceration of edematous, nonreducible sigmoid colon. A sigmoid colectomy and upper rectum resection (Hartmann’s procedure) were performed with the creation of a terminal descending colostomy in the left lower quadrant. There was no additional vaginal vault prolapse noted following sigmoid colectomy and replacement of bowel contents. A 3 cm vaginal vault dehiscence was repaired, and a small perineorrhaphy was performed. At the end of the procedure, the vaginal vault was noted to be well-supported.

## 6. Postoperative Course

The patient was admitted to the hospital and monitored postoperatively. She tolerated advancement to a regular diet with the return of ostomy function by postoperative day 5. A wound vacuum-assisted closure (VAC) device was placed over the midline incision, and enterostomy nursing provided colostomy education prior to discharge. She was discharged to a skilled nursing facility with a Foley catheter in place after an unsuccessful voiding trial and was instructed to complete a course of antibiotics for her urinary tract infection (UTI). Close interval follow-up was arranged with both general surgery and urogynecology. A repeat retrograde voiding trial was attempted in the outpatient setting two weeks postoperatively, but was again unsuccessful. Subsequently, at her one-month postoperative follow-up, she successfully passed the voiding trial, and the Foley catheter was removed without complication. The patient continues to be closely followed for long-term urgency urinary incontinence for which she had previously failed behavioral and medical management, including Mirabegron, tolterodine, and oxybutynin. Prior to her presentation with the large bowel evisceration, she had experienced some improvement in her urgency urinary incontinence with a combination of onabotulinumtoxinA and solifenacin. However, at her postoperative visits following the bowel evisceration, she preferred to await repeat onabotulinumtoxinA injections until after completion of her colostomy reversal, which will be scheduled next month.

## 7. Pathology

Final histopathological examination revealed three segments of colon with reactive mucosal changes, acute and chronic serositis, pulse granuloma with pill fragments, serosal adhesions, and associated fibroadipose tissue exhibiting fat necrosis and hemorrhage.

## 8. Discussion

Evisceration of abdominal contents following vaginal cuff dehiscence is a rare and life-threatening complication of pelvic surgery. Although slightly over 100 cases of transvaginal small bowel evisceration have been documented, description of large bowel evisceration is limited to a handful of case reports [2,3,4,5,6]. Evidence regarding the contribution of surgical technique is conflicting, with some studies hypothesizing that electrosurgery or suture choice may impact the risk of cuff dehiscence and subsequent evisceration [1]. Transvaginal small bowel evisceration carries a mortality rate of 6–8% and a morbidity rate of 15–20% [7]. In roughly 20% of cases, surgical intervention involving bowel resection and anastomosis, along with repair of the vaginal defect, is required [7]. Due to the rarity of large bowel evisceration, data are currently limited, but all reported cases presented acutely and necessitated emergency surgical intervention [2,4,5,8].

The etiology for sigmoid colon evisceration in this case remains unclear but is believed to be multifactorial. Generally, risk factors for transvaginal small bowel evisceration include older age, abdominal hysterectomy, prior pelvic surgery, and pelvic organ prolapse [1]. Notably, approximately 70% of affected individuals are postmenopausal, with a mean age of 62 years, likely due to the hypoestrogenic state, chronic tissue devascularization, and pelvic floor weakness [6,9]. Although rare, premenopausal women may also be affected, typically in association with vaginal trauma or sexual activity. Other contributing factors include lifestyle, obesity (body mass index (BMI) > 27 kg/m^2^), chronic cough, constipation with straining, vaginal trauma from instrumentation, prior pelvic radiotherapy, endocrine disease such as hypothyroidism, multiparity, and connective tissue disease with poor collagen structure [6]. Although less common, Sinhaa et al. reported on bowel evisceration through a ring pessary [10]. In the present case, the patient’s highest risk factors were postmenopausal status, history of hysterectomy and multiple pelvic surgeries, a body mass index of 37 kg/m^2^, and prior pessary use.

Patients with transvaginal bowel evisceration present acutely with mild to severe abdominal pain or discomfort, with or without nausea or emesis, vaginal bleeding, vaginal prolapse or mass, vaginal discharge, hematuria, urinary retention, and/or infection or peritoneal irritation. The diagnosis and treatment of transvaginal evisceration should be completed in a timely manner. This is a life-threatening condition, and emergent intervention is required to prevent subsequent complications relating to bowel ischemia and infarction and to reduce associated morbidity and mortality [3]. The current report is unique both in the evisceration of the large bowel as well as in its subacute presentation. In this case, the patient reported ongoing worsening prolapse symptoms for approximately 3 weeks with an initially reducible vaginal mass. When ultimately prompted to present to care when the mass became no longer reducible, she remained hemodynamically stable and reported ongoing bowel function without overt symptoms of infection despite her 3-week history of bowel evisceration. It is possible that the patient’s ability to remain relatively well appearing despite her bowel evisceration was secondary to her intravenous antibiotic treatment for her cellulitis. The appearance and size of the sigmoid colon, as opposed to the small bowel, likely delayed the patient’s presentation, as the sigmoid look can be mistaken for the vagina. Serati et al. [2] reported on a patient presenting to her routine six-month postoperative visit after a vaginal hysterectomy who was noted to have vaginal vault prolapse. The patient was taken to the OR for vaginal vault prolapse repair, and intraoperatively, the surgeons recognized that she had sigmoid evisceration. That patient was able to have her sigmoid reduced and replaced abdominally as the sigmoid appeared healthy, and the repair of the vagina was accomplished vaginally [2].

A multidisciplinary approach to transvaginal evisceration is crucial, with evaluation potentially involving urogynecology, general surgery, urology, and an intensivist in septic patients [5]. Some literature states that a combined laparoscopy and vaginal approach may be utilized initially to inspect and reposition the eviscerated bowel with repair of vaginal defect [11]. However, laparotomy combined with transabdominal or transvaginal vault repair may be required if a segment of bowel is nonreducible, ischemic, and non-viable, requiring resection and reanastomosis or the creation of an end-colostomy (Hartmann procedure) as in this case [5]. The surgical approach should be individualized, as there is no unified consensus on the optimal surgical technique [6].

## Data Availability

The original contributions presented in this study are included in the article. Further inquiries can be directed to the corresponding author.
